# Supplementary material for: The interactome of intact mitochondria by cross-linking mass spectrometry provides evidence for coexisting respiratory supercomplexes
Source: Mol Cell Proteomics. 2017 Dec 8;17(2):216–32. doi: 10.1074/mcp.RA117.000470 (PMC5795388; doi:10.1074/mcp.RA117.000470)
Supplement: Supplemental Data [file supp_RA117.000470_133922_0_supp_23978_fzffwf.zip › spectra_annotation/mito_spectra_annotation/414-1-12-1-9-1.pdf]

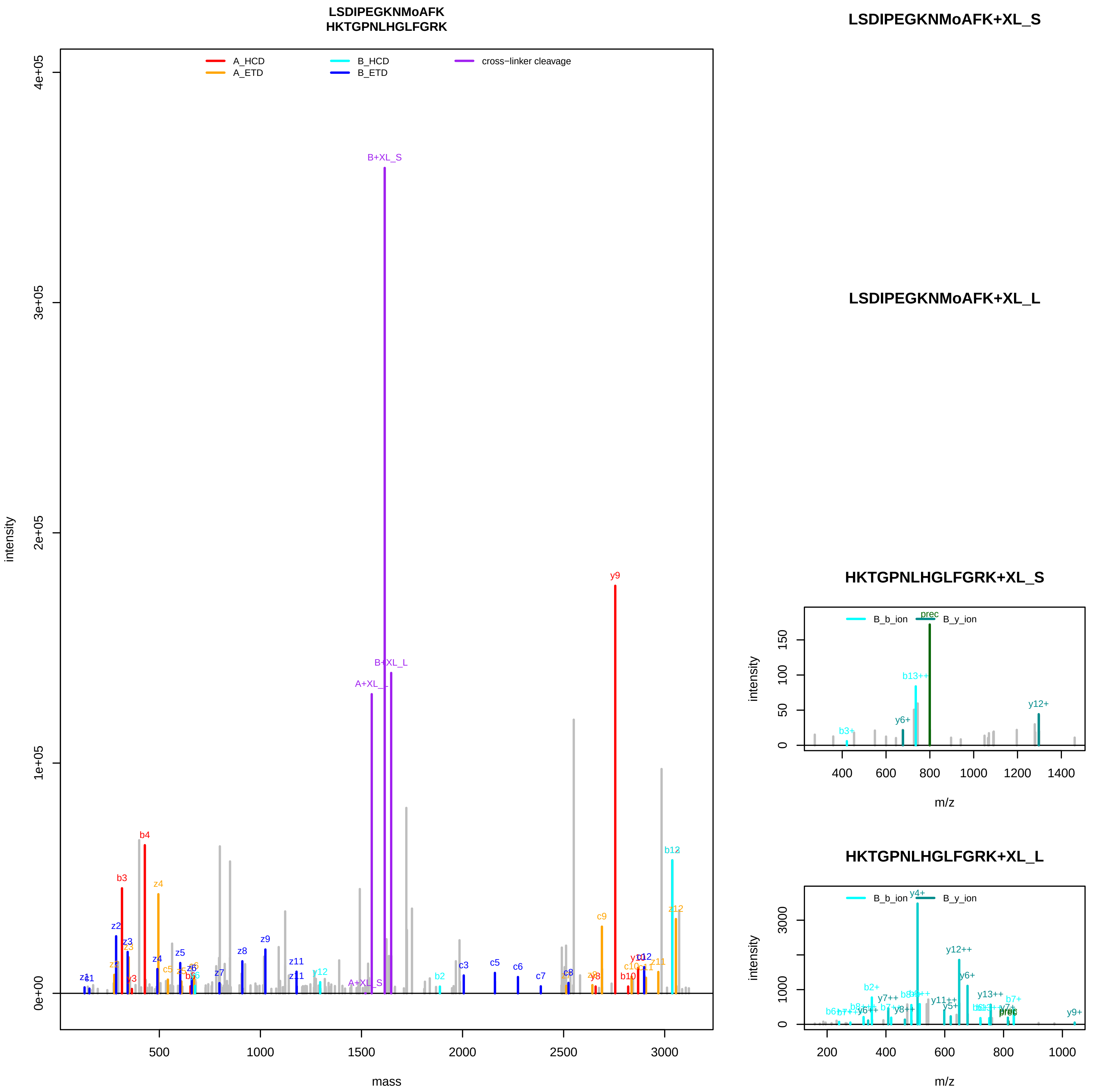

LSDIPEGKNMoAFK
HKTGPNLHGLFGRK
A_HCD
A_ETD
B_HCD
B_ETD
cross−linker cleavage
B+XL_S
B+XL_L
A+XL_L
A+XL_S
y9
b12
z12
c9
b4
b3
z4
z2
z3
z9
z8
z5
z11
c3
c5
c6
c7
c8
b2
y12
b10
c10
c11
z11
intensity
mass
0e+00
1e+05
2e+05
3e+05
4e+05
500
1000
1500
2000
2500
3000
LSDIPEGKNMoAFK+XL_S
LSDIPEGKNMoAFK+XL_L
HKTGPNLHGLFGRK+XL_S
B_b_ion
B_y_ion
prec
b13++
y6+
y12+
b3+
intensity
m/z
HKTGPNLHGLFGRK+XL_L
B_b_ion
B_y_ion
y4+
y12++
y6+
b2+
y7++
y11++
y13++
y5+
b7+
y9+
intensity
m/z
